# An Efficient Predictive Model for Myocardial Infarction Using Cost-sensitive J48 Model

**Published:** 2017-05

**Authors:** Atefeh DARAEI, Hodjat HAMIDI

**Affiliations:** Dept. of Information Technology, Faculty of Industrial Engineering, K.N. Toosi University of Technology, Tehran, Iran

**Keywords:** Myocardial infarction, Heart disease, Metacost, Cost-sensitive J48, Weight by relief

## Abstract

**Background:**

Myocardial infarction (MI) occurs due to heart muscle death that costs like human life, which is higher than the treatment costs. This study aimed to present an MI prediction model using classification data mining methods, which consider the imbalance nature of the problem.

**Methods:**

We enrolled 455 healthy and 295 myocardial infarction cases of visitors to Shahid Madani Specialized Hospital, Khorramabad, Iran, in 2015. Then, a hybrid feature selection method included Weight by Relief and Genetic algorithm applied on the dataset to select the best features. After selection of the features, the metacost classifier applied on the sampled dataset. Metacost made a cost sensitive J48 model by assigning different costs ratios for misclassified cases; include 1:10, 1:50, 1:100, 1:150 and 1:200.

**Results:**

After applying the model on the imbalanced dataset, the cost ratio 1:200 led to the best results in comparison to not using feature selection and cost sensitive model. The model achieved sensitivity, F-measure and accuracy of 86.67%, 80% and 82.67%, respectively.

**Conclusion:**

Experiments on the real dataset showed that using the cost-sensitive method along with the hybrid feature selection method improved model performance. Therefore, the model considered a reliable Myocardial Infarction prediction model.

## Introduction

Acute Coronary Syndrome (ACS) is a type of ischemic heart diseases that include Myocardial Infarction (MI). It occurs when the coronary arteries, narrowed by atherosclerosis, abruptly decrease the blood flow (1). Cholesterol and lipid sediment progressively collects throughout the arteries. This commonly occurs in individuals with genetic susceptibility to atherosclerosis, high blood pressure, an inactive lifestyle, and in those who are overweight or have obesity. The areas with sediment build-up experience the growth of fibrous tissue and calcification, causing the formation of lipid atherosclerosis plaques. Atherosclerosis plaques reduce lumens in the vessels and diminish or block blood flow (2).

MI occurs immediately after creation of thrombosis at sites previously afflicted with atherosclerosis. When coronary artery blood flow ceases after blockage, a small amount of blood will collect in the surrounding vessels. This process is known as MI (1–2). MI is considered as a main cause of death worldwide (3). Cardiovascular diseases are responsible for 30% of deaths worldwide (4) and 40% of deaths in Iran. Among the cardiovascular diseases, MI, commonly known as heart attack (5), is the most common (6).

Data mining is the process of exploring the patterns and knowledge from large datasets (7). Cla**ss**ification, in data mining, finds models that predict the class label for data and predict labels for unclassified data to distinguish the data belonging to each class (8). Traditional classification algorithms decrease classification error by placing instances in true classes.

Heart disease prediction models using data mining methods, such as K-NN (9), SVM (10), ANN (11), NB (12), Decision Tree (13), and Random Forest (14) have been ran a lot, but the researches in predicting MI is very limited. A model was proposed utilized neural network for predicting acute MI in patients, who referred to emergency with chest pain (15). The dataset consisted of 2204 and 40 features, in which 1843 cases had not experienced MI. Finally, sensitivity and specificity of the model were obtained 94.5% and 95.9%, respectively. A c4.5 tree was used for predicting different types of heart diseases, such as MI (16). The data used in this study were the information obtained from 1200 cases, in 416 cases had MI. A c4.5 decision tree was used, rule set classification, Neuro-Fuzzy, Bayesian Network, SVM and time series modeling to predict MI (17). The accuracy and sensitivity of MLP were achieved 89.7% and 90.17%, respectively. In addition, four algorithms were used, namely Naïve Bayes, Decision Tree, MLP and Rule-based Classification, to predict heart disease (18). They applied the algorithms on a heart disease dataset from UCI repository. The best accuracy is obtained using Naïve Bayes that is equal to 84.14%. Masethe and masethe (19) utilized five classification algorithms, J48, Bayesian Network, Naïve Bayes, Classification and Regression Tree and REPTREE, for predicting MI. The data used for this study included 90 MI cases and 18 without MI. After comparing the results, J48, NB, and CART achieved an accuracy of 99.07%. An ECG classification model was proposed for detecting MI in (20). Two methods, SVM and MLP were applied to data. The accuracies obtained for SVM and MLP were 90.17% and 82.14%, respectively. A model was presented in (21) for detecting MI and location, which uses K-NN and SVM which is applied to PTB dataset. The dataset included 290 cases, where 148 of them had MI. Accuracy, sensitivity, and specificity for SVM were 96%, 93% and 99%, respectively.

Traditional classification methods obtained proper accuracy, but they have been applied on almost balanced datasets. In these methods, the number of cases with disease is equal to the number of healthy cases and even more than healthy cases. Generally, if the ratio of smaller class to prevailing class is 1:100, 1:1000 or larger, it can be considered as an imbalance problem (22). Since the results in data mining prediction problems tend to larger classes influenced by the prevailing class, the results of these predictions cannot be considered appropriate. The cost sensitivity was not considered in the models, but for MI prediction, misclassification of a healthy instance only entails additional laboratory costs or angiography side effects, while misclassification of an MI case as healthy could incur costs that involve missing the opportunity for timely use of medicines and treatments and even loss of life. Therefore, the contribution of this study is considering the imbalanced nature of MI dataset using a cost-sensitive classification model to predict MI. Moreover, a hybrid feature selection method, which uses a weighting method and Genetic algorithm along with the cost sensitive model are another considered the other contribution, to make more improvement in the performance. A cost-sensitive model has not been presented for prediction of MI in previous works. The goal of the present study was to determine how a cost-sensitive model could be constructed and employed for MI prediction.

In this study, we proposed a model included a hybrid feature selection method and a cost-sensitive model. The operator Weight by Relief gives weights to the features. Then, top weighted features selected and gave to GA to select the best final features. After turning the dataset to an imbalanced dataset, based on the statistics, the Metacost classifier with embedded J48 decision tree, used to predict MI. Finally, the analysis of the results based on the evaluation measures showed the power of the proposed model. The advantage of the proposed model was the consideration of the cost of misclassification.

## Materials and Methods

### Data Description

Dataset obtained from Shahid Madani Specialized Hospital of Khorramabad, Iran, in 2015. This dataset included the information obtained from750 patients of the mentioned hospital, in which 295 cases were patients with MI and 455 cases were healthy. Dataset included 92 regular features and 1 label feature. These features were demographic, examinations, symptoms, laboratory tests, main coronary arteries, and ECG features, namely Age, Body Mass Index (BMI), Sex, Hypertension (HTN), Diabetes (DM), Smoking, Family History (FH), Obesity, Chronic Renal Failure (CRF), Cerebrovascular Accident (CVA), Thyroid, Airway disease, Hyperlipidemia (HLP), Troponin I, C-reactive protein (CRP), Total Cholesterol, White Blood Cells (WBC), Congestive Heart Failure (CHF), Fasting Blood Sugar (FBS), Creatinine (Cr), Lactate dehydrogenase (LDH), Creatine phosphor-kinase (CPK), Triglyceride (TG), Low-density Lipoprotein (LDL), High-density Lipoprotein (HDL), Blood Urea Nitrogen (BUN), Erythrocyte Sedimentation Rate (ESR), Hemoglobin (Hb), Lymphocyte, Platelet, Ejection Fraction (EF), Potassium (K), Sodium (Na), Systolic Blood Pressure (SBP), Diastolic Blood Pressure (DBP), Heart rate, Edema, Fatigue and weakness, Lung rales, Typical Chest-Pain (C.P), Distribution of pain to arms and neck, Dyspnea, Atypical Chest-Pain (C.P), Non-anginal Chest-Pain (C.P), Exertional Chest-Pain (C.P), Left Anterior Descending Artery (LAD), Right Coronary Artery (RCA), Left Coronary Artery (LCA), T inversion leads (I, II, III, avR, avL, avF, V1, V2, V3, V4, V5, V6), ST Depression leads (I, II, III, avR, avL, avF, V1, V2, V3, V4, V5, V6), ST Elevation leads (I, II, III, avR, avL, avF, V1, V2, V3, V4, V5, V6) and Poor R Progression leads (V1, V2, V3, V4, V5, V6). For each ECG-related feature, the leads of the features are considered as separate features.

### Preprocessing

Real data are usually incomplete and inconsistent (8). The data cleaning method used in the present study was handling missing values. Moreover, for data transformation task, normalization method applied to the data, in which features’ values were scaled in a smaller range, like [0, 1].

All procedures performed in studies involving human participants were in accordance with the ethical standards of the institutional and/or national research committee and with the 1964 Helsinki declaration and its later amendments or comparable ethical standards.

### Feature Selection

Feature selection is a popular method for data reduction, in which irrelevant features of the data is removed. Eliminating the redundant features not only results in more efficiency (23) but also simplifies the understanding and interpretation of the problem (8). Hybrid methods might lead to better performance compared to individual methods (24). Using Evolutionary Algorithms for feature selection results in better accuracy of the classification algorithm (25).

In this study, a hybrid feature selection used to achieve the best subset of features in order to improve the performance. Genetic algorithm (GA) is one of the evolutionary algorithms inspired from nature and tries to find optimized solutions for problems (26). GA has an iterative process, which selects the best ones. By applying crossover and mutation operators afterward, a new child population, with the same size, is generated (27). The other part was “Weight by Relief” operator, in Rapidminer software. Weight by Relief assesses the quality of features for their power in recognizing the cases with the same class and different classes, which are adjacent. It measures the relevance between features by calculating the relevance between features and comparing the values of the feature for the nearest example in the same class and in a different class (28).

### J48 Decision Tree

J48 is a simple form of C4.5 decision tree, which is a method for creating a decision tree. In the classification process in decision trees, the unlabeled cases are classified based on the prior trained classified cases. In decision trees, the leaves represent the classes (29–30).

### Cost-sensitive Learning

In the real world, the cost of wrong labeling in some fields, like medical problems, varies for different classes. For example, the cost of false classification of a patient as a healthy person is much more than misclassification a healthy person. Assign costs to classifiers is one of the most effective methods for handling imbalanced datasets (31). In cost-sensitive algorithms, the cost of false classification of a positive instance as negative and the cost of false classification of a negative instance as positive is different (32). Therefore, misclassification cost plays an important role in some critical problems (33).

In cost-sensitive classification, a cost matrix considered, as shown in Table 1 C (0, 1), C (1, 0), C (0, 0) and C (1, 1) were the costs of False Negative, False Positive, True Negative and True Positive, respectively. Cost-sensitive classification used for classification of imbalanced datasets, in which the class with much fewer cases, considered as positive and the other class with much more cases, called negative (34).

**Table 1:** Cost matrix in cost sensitive methods

**Cost Matrix**	**Actual Negative**	**Actual Positive**
Predicted Negative	C(0, 0)	C(0, 1)
Predicted Positive	C(1, 0)	C(1, 1)

### Metacost

Domingo in (35) declares the purpose of Metacost as adapting the traditional cost-sensitive classifiers. The traditional classifiers were error-based, but Metacost gives different costs to them (36). This method merges a traditional algorithm in a process that minimizes the cost. This way, the algorithm is changed to a cost-sensitive algorithm. In these methods usually, higher cost is given to FN compared to FP (37).

### Proposed Methodology

**Fig. 1:**
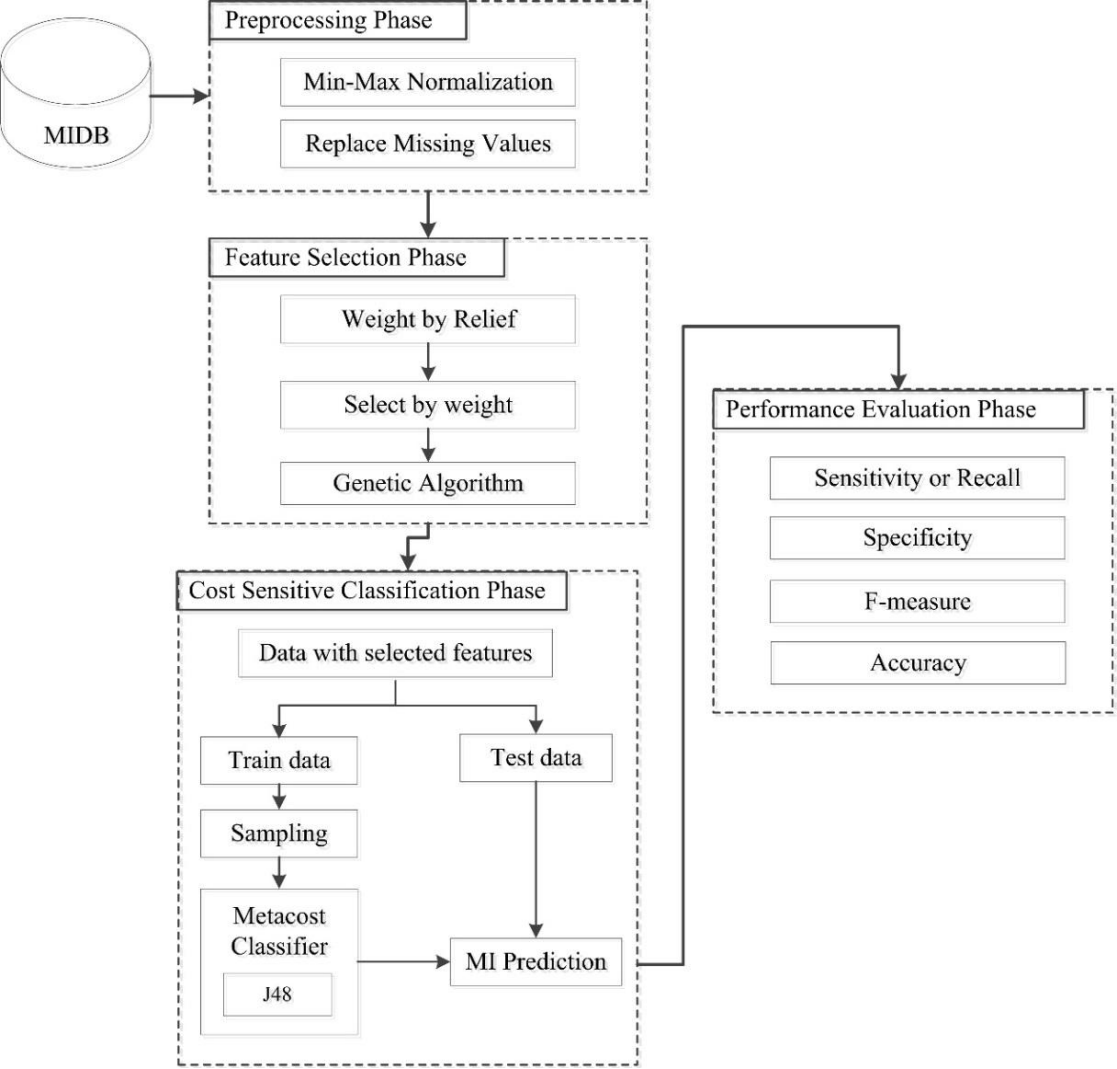
Structure of proposed model

The proposed method employed weight by relief and GA for feature selection. Metacost applied in classification phase, which made J48 cost sensitive (Fig. 1).

#### Phase 1: preprocessing

After collecting the data, the missing values replaced with the average of the other feature values. The data then normalized using min-max normalization (8). The range for normalization was [0, 1].

#### Phase 2: Feature Selection

First, the operator Weight by Relief applied to the normalized features. Then “Top *P*%” selection used at *P*=0.7, meaning that features with weights in the top 0.7 selected. In the second step, GA applied to the features selected in the previous step and selected the final group of best features (Table 2). The method, presented in (38), used to obtain the probability of mutation using the relation 1/(4 × no. of features).

**Table 2:** Parameter setting for GA

Max. number of generations	100
Population size	100
Selection Method	tournament
Mutation probability	0.6
Crossover probability	0.00271
Crossover type	one point
Early stop criteria	No improvement for 10 iterations

#### Phase 3: Cost-Sensitive Classification

For cost-sensitive classification, first, the data divided into training and testing sets. The training data comprised 90% of the data and the remaining 10% used as test data. In Iran, MI occurs at a rate of 14 per 1000 individuals (39). Initially, the MI cases in the dataset were similar to the number of healthy cases; thus, the MI cases sampled at a ratio of 0.014 for MI and 1 for healthy cases. Metacost with embedded J48 then applied for classification. Metacost used to make the J48 algorithm cost-sensitive. For J48 algorithm, the default setting parameters selected in Rapidminer. For Metacost, the maximum number of iterations set to 100. Since the performance of the models is affected by different costs (40), the cost for false positive (FP) set to one, but costs of 10, 50, 100, 150 and 200 considered for false negative (FN).

#### Phase 4: Performance Evaluation

Many measures exist for evaluating classification measures. Accuracy is the ratio of correctly classified cases. It calculated using Equation [1] as:

$$\mathrm{Accuracy}=\frac{\mathrm{TP}+\mathrm{TN}}{\mathrm{TP}+\mathrm{TN}+\mathrm{FP}+\mathrm{FN}}$$

Accuracy was not appropriate for evaluating imbalance datasets (8); thus, sensitivity (or recall), specificity and F-measure (8) used to evaluate Metacost classification performance.

Sensitivity or Recall provides the ratio of positive instances correctly classified (8). This measure is frequently used in the field of medicine to show the rate of correct diagnosis of disease (41). Specificity provides the ratio of negative instances that are correctly classified and demonstrates the tendency to detect healthy instances. Sensitivity and specificity obtained using Equations [2] and [3] as:

$$\mathrm{Sensitivity}=\frac{\mathrm{TP}}{\mathrm{TP}+\mathrm{FN}}$$

$$\mathrm{Specificity}=\frac{\mathrm{TN}}{\mathrm{TN}+\mathrm{FP}}$$

The F-measure is the average of the recall and precision measures. It obtained using Equation [4] as:

$$F-\mathrm{measure}=\frac{2*\mathrm{precision}*\mathrm{recall}}{\mathrm{precision}+\mathrm{recall}}$$

Which precision obtained using Equation [5] as:

$$\mathrm{Precision}=\frac{\mathrm{TP}}{\mathrm{TP}+\mathrm{FP}}$$

## Results

### Experimental Results

Rapidminer (ver. 7.1.001) used to implement the model. After selecting the top 70% of the data, this operator selected 64 features. GA then selected the 62 best final features namely Troponin I, ST Elevation I, ST Elevation avL, ST Elevation avF, ST Elevation III, ST Elevation II, ST Elevation V2, ST Elevation V4, ST Elevation V3, ST Elevation V1, T inversion V3, T inversion III, T inversion V2, T inversion avF, T inversion V6, T inversion avL, T inversion V5, T inversion V1, T inversion V4, T inversion II, T inversion I, Poor R Progression V4, Poor R Progression V2, Poor R Progression V3, ST Depression V3, ST Depression V2, ST Depression V1, ST Depression III, ST Depression avR, ST Depression V4, ST Depression avF, Non-anginal C.P, HLP, Distribution to arms and neck, SBP, Typical C.P, RCA, LAD, LCX, DM, Sex, EF, FH, Lymphocyte, Dyspnea, LDL, TG, Fatigue and weakness, Smoker, Atypical C.P, HDL, LDH, Age, Lung rales, CRP, BUN, Total cholesterol, HTN, Exertional C.P, CPK, DBP.

**Fig. 2:**
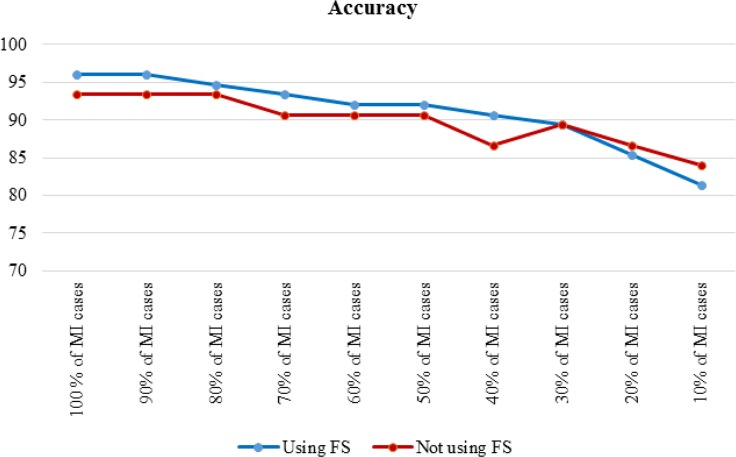
Accuracy of cost insensitive J48 in decreasing MI cases

**Fig. 3:**
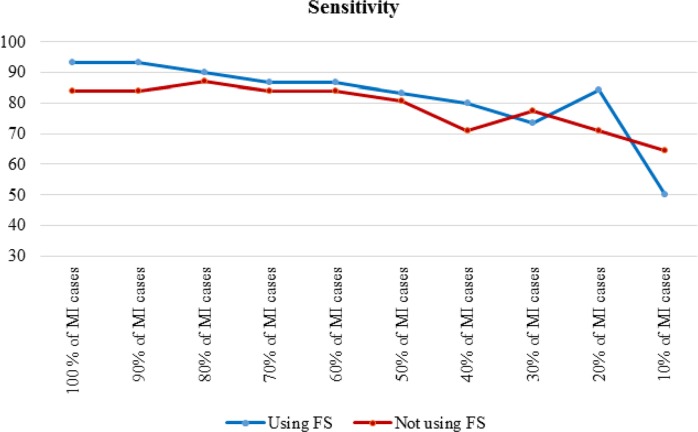
Sensitivity of cost insensitive J48 in decreasing Mi cases

To assess the effect of the size of the positive class on model performance, the results of the J48 decision tree for the cost-insensitive state presented. In this case, all healthy cases used, but the number of MI cases gradually reduced. Table 3 shows the results of cost insensitive J48 decision tree, before and after feature selection. For easier understanding, the accuracy and sensitivity, respectively, during the decrease for the dataset are shown in Fig. 2, 3.

**Table 3:** Performance of cost insensitive J48 in decreasing MI cases

	**No FS**			**FS**		
	**Accuracy (%)**	**Sensitivity (%)**	**Specificity (%)**	**Accuracy (%)**	**Sensitivity (%)**	**Specificity (%)**
100 % of MI cases	93.33	83.87	100	96	93.33	97.78
90% of MI cases	93.33	83.87	100	96	93.33	97.78
80% of MI cases	93.33	87.10	97.73	94.67	90	97.78
70% of MI cases	90.67	83.87	95.45	93.33	86.67	97.78
60% of MI cases	90.67	83.87	95.45	92	86.67	95.57
50% of MI cases	90.67	80.65	97.73	92	83.33	97.78
40% of MI cases	86.67	70.97	97.73	90.67	80	97.78
30% of MI cases	89.33	77.42	97.73	89.33	73.33	100
20% of MI cases	90.67	80.65	97.73	84	66.67	95.56
10% of MI cases	84	64.52	97.73	80	50	100

**Fig. 4:**
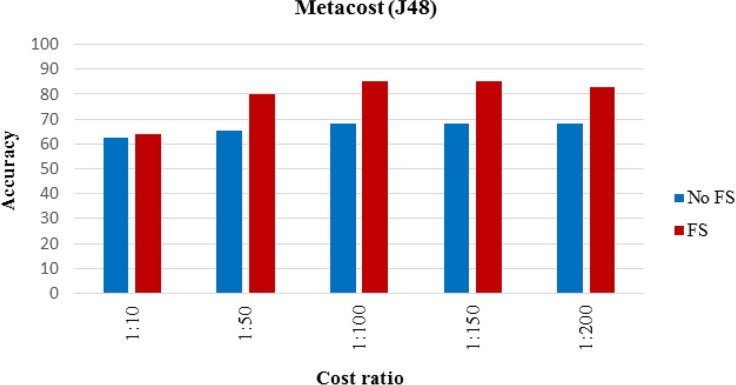
Accuracy of proposed cost sensitive model

**Fig. 5:**
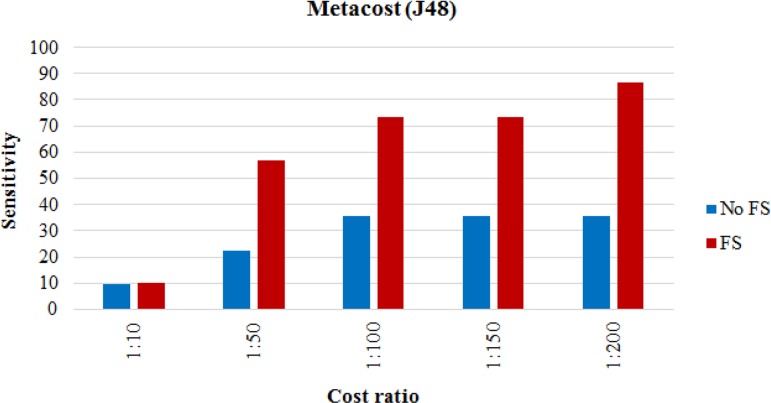
Accuracy of proposed cost sensitive model

**Fig. 6:**
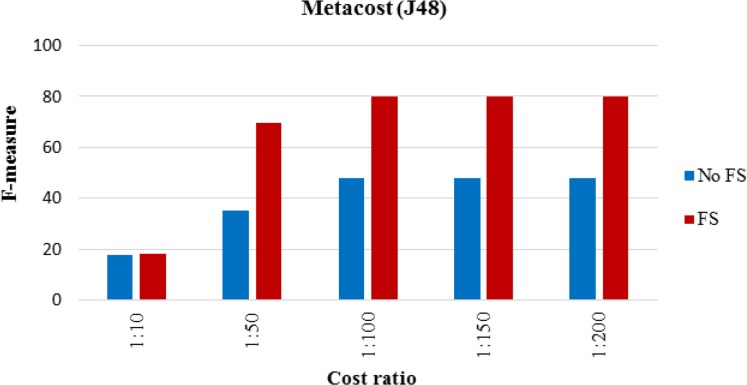
Accuracy of proposed cost sensitive model

The proposed MI prediction model tested for the new dataset in which there were 7 MI cases. Table 4 shows the results of performance analysis of model implementation. Although accuracy was not the main performance criterion, the other criteria’s results also provided. Fig. 4–6 show the trends for sensitivity and F-measure before and after feature selection.

**Table 4:** The results of the proposed cost sensitive J48 model

		**Cost ratio (cost of FN: cost of FP)**					
		**No costs**	**1: 10**	**1: 50**	**1: 100**	**1: 150**	**1: 200**
Accuracy	No FS	58.67	62.67	65.33	68	68	68
	FS	60	64	80	85.33	85.33	82.67
Sensitivity	No FS	0	9.68	22.58	35.48	35.48	35.48
	FS	0	10	56.67	73.33	73.33	86.67
Specificity	No FS	100	100	95.45	90.91	90.91	90.91
	FS	100	100	95.56	93.33	93.33	80
F-measure	No FS	-	17.65	35	47.83	47.83	47.83
	FS	-	18.18	69.39	80	80	80

## Discussion

Feature selection improved performance improvement; however, Fig. 2 and 3 indicated that the effect of feature selection diminished, as the number of positive cases decreased. The accuracy and sensitivity of the model were higher than feature selection when 20% and 10% of the MI cases were used, respectively. The effect of feature selection was evident after the addition of the cost-sensitive J48 algorithm. Table 3 indicated that increasing the cost of FN improved performance. Assigning a cost to FN improved the accuracy of J48 at least 4% before and after feature selection. Assigning a cost to FN improved the sensitivity of J48 at least 10%. The model achieved a high F-measure score by increasing the cost, which indicated that both the precision and robustness of the model increased.

Feature selection combined with a cost-sensitive model significantly improved the accuracy, sensitivity, and F-measure. A high specificity score was desirable, but an increase in costs decreased specificity. Although the increase in costs for FN improved performance, increasing the costs did not always improve the performance. In implementation of J48, after assigning a cost ratio of 1:250 to the model, the sensitivity, specificity and accuracy scores were 100%, 0%, and 40%, respectively. In this case, despite very appropriate sensitivity, the specificity was zero, which cannot be considered good performance of the model. Based on the importance of trade-off between sensitivity and specificity, the implementation of cost-sensitive J48 at a cost ratio of 1:200 provided the best model. At last, the cost-insensitive models could not predict MI cases in the imbalanced datasets, while the appropriate sensitivity of the proposed model indicated satisfactory prediction. A limitation of the present study was the unavailability of the features of Q-wave and Rhythm in the dataset. Future research will expand the model with hybrid classification algorithms.

## Conclusion

Feature selection improved the performance of both cost-insensitive and cost-sensitive models. Moreover, making J48 cost-sensitive improved performance over traditional classifiers and achieved a better trade-off between sensitivity and specificity. The advantage of the present model is enhancement of sensitivity for MI prediction, which means the model has higher tendency to predict MI cases correctly.

## Ethical considerations

Ethical issues (Including plagiarism, informed consent, misconduct, data fabrication and/or falsification, double publication and/or submission, redundancy, etc.) have been completely observed by the authors.
